# Impact of formal support services on care recipient behavioral symptoms of dementia and care partner burden

**DOI:** 10.1002/alz70858_105325

**Published:** 2025-12-26

**Authors:** Adam Mohammed, Ishan Canty Williams, Meghan K Mattos

**Affiliations:** ^1^ University of Virginia, Charlottesville, VA, USA

## Abstract

**Background:**

Caring for older adults with dementia in a community is preferred by care recipients and helps reduce the high cost of institutional care. Community‐based care is often by unpaid or informal care partners (CPs) and can burden CPs and increase their risk for poor outcomes. While evidence shows that support services may benefit older adults with dementia and informal CPs, their impact on CP burden and care recipient behavioral concerns remains unclear. The aim of this scoping review was to synthesize evidence on the effects of support services or interventions on CP burden and care recipient behavioral concerns.

**Method:**

A literature search was conducted in PubMed, CINAHL, and EMBASE for studies published from 2014 to September 2024. The search strategy included keywords covering dementia caregiving, support services or intervention, caregiver (care partner) burden, and behavioral or psychological symptoms of dementia. Inclusion criteria included original studies describing the effects of support services or interventions on subjective CP burden and care recipient behavioral or psychological symptoms of dementia. Studies not conducted in the US or involving care recipients in care facilities were excluded. Data were extracted, organized in tables, compared, and synthesized.

**Result:**

The search yielded 923 articles screened by title and abstract. Eighty‐six full‐text articles were reviewed, and 12 met inclusion criteria. Articles presented quasi‐experimental (*n* = 7) or randomized controlled trial (*n* = 5) designs. Support services or interventions included care management programs (*n* = 4), behavioral training (*n* = 3), psychoeducational programs (*n* = 3), and support groups (*n* = 2). Among the 12 support services, *n* = 6 targeted care recipients and CPs, *n* = 5 targeted only CPs, and n =1 targeted only care recipients. Seven of the 12 articles reported a significant reduction in CP burden after support service participation: four reported significant reductions in the frequency and severity of care recipients’ behavioral concerns. Most effective interventions (*n* = 4 of 7) were delivered in person compared with remote methods.

**Conclusion:**

Findings suggest a variety of support services, especially when delivered in person, can help alleviate CP burden and care recipient behavioral concerns. This underscores the need for further dyadic research to support dementia patients and their CPs in community settings.

